# The Food Addiction Clinical Treatment (FACT) Manual: A Harm Reduction Treatment Approach

**DOI:** 10.3390/bs14070557

**Published:** 2024-07-01

**Authors:** Erin L. O’Hea, Shenelle A. Edwards-Hampton, Dana L. Beall Brown, Kendrin R. Sonneville, Douglas M. Ziedonis, Ashley N. Gearhardt

**Affiliations:** 1 Department of Psychology, Stonehill College, North Easton, MA 02357, USA; 2 Department of General Surgery, Wake Forest University School of Medicine, Winston-Salem, NC 27157, USA; saedward@wakehealth.edu; 3 Department of Psychiatry, University of Colorado School of Medicine, Aurora, CO 80045, USA; dana.beallbrown@cuanschtuz.edu; 4 Department of Nutritional Sciences, University of Michigan School of Public Health, Ann Arbor, MI 48109, USA; kendrins@umich.edu; 5 Department of Psychiatry and Behavioral Science, University of New Mexico, Albuquerque, NM 87131, USA; dziedonis@salud.unm.edu; 6 Department of Psychology, University of Michigan, Ann Arbor, MI 48109, USA; agearhar@umich.edu

**Keywords:** food addiction, clinical treatment, harm reduction, cognitive behavioral therapy

## Abstract

While the construct of food addiction has been controversial, there is growing evidence that certain foods can activate biobehavioral and neurological mechanisms consistent with addiction to other substances. Despite increased evidence and acceptance of certain foods as addictive substances amongst the scientific community, there is a paucity of interventions available that are uniquely suited for the treatment of this condition. Further, many of the addiction and disordered eating treatment models currently utilized for food addiction are seemingly at odds, with the former often recommending complete abstinence from trigger foods and the latter promoting intake of all foods in moderation. The Food Addiction Clinical Treatment (FACT) manual was created as an alternative using an empirically supported harm-reduction model specifically targeted to treat the addiction and disordered eating features of food addiction. The purpose of the current article is to expose readers to the key tenets of the FACT manual, demonstrate the feasibility of this intervention with a sample of participants with severe food addiction, and discuss future directions for the treatment of food addiction. Positive outcomes from this intervention provide preliminary evidence for the efficacy of FACT for the treatment of food addiction with minimal negative adverse effects. Future research using randomized control trials and longer follow-up is needed to validate the FACT manual as an empirically supported treatment for food addiction.

## 1. Introduction

Research is growing that addictive processes may contribute to certain types of eating-related problems [1]. Behavioral indicators of addiction are common in eating problems, such as loss of control over consumption, continued use despite negative consequences, intense cravings, inability to reduce intake, and high rates of relapse [2]. The neural systems that contribute to addiction are also implicated in obesity and overeating, such as hyperactivation of the mesolimbic dopaminergic system to relevant cues [3]. Importantly, not all foods hold the same potential for triggering an addictive response [1]. Ultraprocessed, hyperpalatable foods with high levels of added fats and refined carbohydrates have been linked with behavioral and biological changes associated with addiction in animal models, and have been identified as having higher risk for activating an addiction-response in some humans [1,2,4].

The Yale Food Addiction Scale (YFAS) is the most commonly utilized measure of addictive eating and applies the diagnostic indicators of substance use disorders to the intake of hyperpalatable ultraprocessed foods (e.g., chocolate, soda, pizza) [5]. The most recent version of this measure (i.e., YFAS 2.0) assesses for the frequency of 11 diagnostic criteria for addiction (e.g., loss of control over intake, intense cravings, inability to cut down, withdrawal, tolerance), as well as clinically significant impairment/distress, over a 12-month period [6]. Akin to a substance use disorder diagnosis, food addiction is designated by the presence of two or more symptoms plus clinically significant impairment/distress in the past year. Food addiction and existing eating disorders are related but distinct constructs, in both phenotypic presentation and theoretically relevant mechanisms, and most individuals with food addiction do not meet criteria for an existing eating disorder diagnosis [1]. Addiction can occur in patterns that are not consistent with binging behavior. For example, in the case of chain smoking, use of the substance is continuous (does not occur within a discrete period of time) and is not typically accompanied by a subjective sense of loss of control. Further, mechanisms like reward dysfunction, withdrawal, and tolerance are causally implicated in addictive disorders but not eating disorders [7]. Further, models of addiction s highlight the importance of the highly rewarding substance (e.g., cigarettes, alcohol) or behavior (e.g., gambling) in driving forward patterns of compulsive intake [7].

Although food addiction is distinct from eating disorders and obesity, these conditions may co-occur. A recent meta-analysis based on 272 global studies estimated that 14% of adults in nonclinical samples met criteria for a “diagnosis” of food addiction with higher levels in clinical samples with obesity (28%) and binge eating disorder (55%) [5]. While food addiction is not currently an officially recognized diagnosis, there is growing scientific interest in its clinical utility. A recent large-scale treatment trial (the DIETFITS Trial) for obesity found that food addiction was the strongest psychosocial predictor of treatment response failure [8]. For individuals with binge-type eating disorders, food addiction is associated with more severe psychopathology and a greater likelihood of drop out from traditional treatment approaches such as psychoeducation interventions and cognitive behavioral therapy (CBT) [9,10]. Co-occurring food addiction may present unique treatment challenges due to its association with higher levels of impulsivity, emotion dysregulation, and a lower quality of life across multiple domains [11].

In the context of co-occurring food addiction and eating disorders, significant dissonance in the interventions most commonly used for the treatment of disordered eating versus the treatment of addiction presents a unique challenge to treatment. Standard approaches to treating loss-of-control disordered eating episodes (e.g., CBT for binge eating disorder; CBT for BED), emphasize the role of harmful cognitions and maladaptive dietary behaviors in the onset and course of this condition [12]. For example, CBT for BED focuses on restructuring rigid dietary beliefs, challenging body shape and weight concerns, and reducing episodes of food restriction [12]. While this approach does address several mechanisms associated with addiction (like the development of skills to manage cravings and the identification of cues that trigger binge eating episodes), it does not acknowledge the potentially addictive nature of the hyperpalatable, ultra-processed foods that are the predominantly consumed in binges [1,7]. On the contrary, these treatments often encourage continued intake of foods with high addictive potential, discouraging the restriction of any particular food from one’s diet with the goal of limiting rigid or binary (e.g., “good” vs. “bad” foods) food categorization that is often taken to an extreme among individuals with eating disorders [12,13]. Given this incongruity, it is likely that most individuals with food addiction are not being adequately assessed, diagnosed, or treated under traditional eating disorder models [1].

Current CBT-based eating disorder treatment approaches stand in contrast with some of the most widely used treatments for addiction, which promote complete abstinence from the addictive substance, such Alcohol Anonymous (AA) and other 12-step treatment programs. To make matters even more complex, there are several popular community-based treatment programs that have applied abstinence-based addiction models to the treatment of loss-of-control eating (e.g., Overeaters Anonymous, Food Addicts Anonymous) [14]. However, these abstinence-based treatment models have received little empirical study and concerns have been raised that they may lead to greater eating disorder pathology in some individuals [14,15]. Further, the current food supply in much of the world is predominantly composed of hyperpalatable, ultraprocessed foods [16], which may make completely avoiding these foods unattainable for many. Thus, there is a growing need to develop empirically supported interventions for the treatment of food addiction. For those vulnerable to addictive-like eating, the ability to control consumption of hyperpalatable, ultra-processed foods and eat them moderately may be extremely challenging. This may be even more difficult for individuals from a disadvantaged background. However, trying to rigidly abstain from all high-risk foods may evoke feelings of deprivation and trigger out-of-control eating. In the field of addiction, a personalized plan of moderated substance use is an empirically supported approach that does not require abstinence, but aims to help people consume addictive substances safely (i.e., harm reduction) [17]. Harm reduction approaches offer an alternative to abstinence-based approaches and seek to minimize the negative consequences that are commonly associated with addictive substance intake. While harm reduction approaches were initially highly controversial in the addiction field, empirical evidence has supported their utility as an effective treatment intervention [18,19]. A harm reduction treatment approach may also provide a useful treatment framework for food addiction that both addresses the addictive potential of ultraprocessed foods, while not requiring complete abstinence from broad and prevalent categories of the food supply. However, no harm-reduction-based treatment for food addiction has been documented.

The current study aims to provide theoretical and practical foundations for the application of a harm reduction treatment modality to food addiction. Given that individuals with food addiction do not appear to respond as effectively to traditional psychosocial treatments for binge-type eating disorders or obesity [8,9], it is essential that novel interventions are developed that address addictive mechanisms without causing adverse consequences. We will first discuss the content of a harm-reduction based treatment manual (Food Addiction Clinical Treatment (FACT)) developed by an interdisciplinary team of clinicians and scientists with expertise across the domains of food addiction, substance addiction, eating disorders, obesity, and nutrition. Next, we describe the results for an initial acceptability and feasibility trial of two participants with severe food addiction as defined by the YFAS. Specifically, we investigate the relationship between participation in FACT and food addiction symptomology, quality of life, self-efficacy, and levels of depression and anxiety. Finally, we will evaluate whether participation in FACT resulted in unintended adverse consequences, such as greater disordered eating and unhealthy restrictive practices (e.g., fasting, purging, laxative use, compulsive exercise), increases in internalized weight bias, or a transfer to increased problematic intake of other addictive substances. This provides the first step for the further evaluation of harm reduction treatment modalities as a potential approach for treating food addiction.

## 2. FACT Manual

### 2.1. Fact Manual Goals

The goals for individuals receiving this treatment are as follows:

1. To understand the symptoms of food addiction within a harm-reduction context;
2. To identify individualized triggers (e.g., people, places, things, emotions, situations, etc.) that play a role in the consumption of high-risk hyperpalatable foods;
3. To identify foods that are personally higher-risk and learn how to reduce harm of identified foods;
4. To improve awareness of mindless eating patterns, cravings for high-risk foods, and how to employ mindful eating approaches to reduce harm;
5. To gain skills related to: eating/preparing foods that are lower-risk for addictive processes, meal planning, coping with cravings for high-risk foods, and coping with negative emotions and stress without food
6. To develop a personalized post-treatment plan, regarding high-risk processed and low-risk naturally occurring foods, that is either moderation or abstinence-based, depending on the participant ’s goals and preference

### 2.2. FACT Manual Session Structure

- Check-in and homework discussion: Individuals are invited to share their current emotional status and difficulties/successes they experienced related to food addiction since the last session. Homework from the past week is reviewed and participants are provided with feedback.
- Psychoeducation: Individuals are provided with research-informed weekly didactics related to food addiction and skill implementation for the treatment of addiction and disordered eating behaviors.
- Group or buddy exercises: Skills taught in each session are practiced with peers in the group or amongst the group as a whole.
- Homework assignment: Individuals are asked to complete weekly journaling exercises and handouts for continued skill development prior to the next treatment session.

### 2.3. FACT Manual Content

The FACT manual is separated into four phases. The first phase is titled *Understanding Food Addiction*. Phase 1 is dedicated to psychoeducation regarding food addiction and general addiction processes, as well as the abstinence versus harm reduction models. This first phase also seeks to increase understanding of personalized addiction to specific foods (i.e., identifying personal triggers and high-risk situations and foods). 

Phase 2 is *Skill Building* and provides a deeper dive into risky situations; coping strategies; the interaction between cognitions, emotions, and behaviors; and the impact of relationships and communication on food addiction. Unique skills are taught in this domain relevant to empirically supported treatments for addiction. For example, similar to established addiction treatments, this section discusses cognitive expectancies about the effect of certain foods (e.g., increasing pleasure, reducing negative effect) that can activate food intake, even if the food is no longer providing those benefits [20,21]. Identifying and challenging positive expectancies about high-risk foods (typically hyperpalatable, ultraprocessed food) is also part of this phase of treatment, as is empirically supported CBT skills. For example, group members are provided psychoeducation regarding the relationship between cognitions, emotions, and behaviors and the powerful positive or negative feedback loop that can occur as a result of an individual’s self-statements, particularly with respect to eating patterns. Content is intended to teach group members how to identify faulty thought patterns and how to restructure problematic thoughts to be more accurate, realistic, and balanced with the goal of positively impacting behaviors with food. A description of the skills covered in this phase can be found in Supplementary Table S1.

In addition to a range of skills, this phase also introduces a clinical tool, created specifically for the FACT manual, called the Food and Eating Addictions Test (FEAT). The FEAT helps individuals identify personalized risk level for foods in a given context. The FEAT allows each group member to calculate the overall risk of specific foods based on ratings of personal and situational risk, on a 1 to 9 scale (Supplementary Figure S1). This tool also prepares individuals for an upcoming intervention in Phase 3 of treatment.

Phase 3 is *Hitting the Reset Button*, which consists of preparing and engaging in the “Lifestyle Launch” (LL). The LL involves 4 weeks of detoxification from the participant’s personalized high-risk foods and a general detoxification from hyperpalatable ultra-processed foods. Research has identified that beneficial neural adaptations can occur after four weeks of alcohol abstinence, including altered neural connectivity in frontoparietal networks involved in top-down control of impulsivity [22] and reduced reward-related neural responsivity to alcohol cues [23]. Phase 3 LL was informed by this literature and individuals are asked to take a four-week break from foods that they identify as high-risk (e.g., hyperpalatable ultraprocessed foods) and to primarily consume foods that they identify as low-risk (e.g., minimally processed, whole foods). During Phase 3, continued psychoeducation about principles of relapse prevention (e.g., lapse versus relapse) and skill building (e.g., meal planning, dining out, mindful eating) are provided. Personalized goals for the next stage of treatment are also discussed (i.e., continuing to take a break from high-risk foods or adding back in moderate-risk foods). 

Finally, Phase 4 follows the LL and is labeled *Exposure and Maintenance*. During this phase, group members are exposed to higher-risk foods and situations to increase their confidence and ability to manage real-life situations. Phase 4 also involves personalized planning for the future and continued development of relapse prevention skills consistent with harm-reduction treatment approaches [17,24]. Finally, the treatment concludes with a “graduation” for group members as well as individualized referrals for those in need of continued mental health resources.

The FACT manual was written to a 6th grade reading level. The material present in each topic in the intervention was reviewed by experts in the fields of addiction treatment, disordered eating, and obesity management for fit within a harm reduction framework to treating food addiction. A full outline of the FACT manual content can be found in Supplementary Table S1.

## 3. Pilot Study Methods

### 3.1. Procedure

#### 3.1.1. Recruitment and Enrollment

Participants were recruited to participate in a treatment trial for food addiction via physical and electronic advertising at a large local academic medical center. Interested participants completed a pre-screener to determine eligibility for participation in the clinical treatment. Eligible participants endorsed clinically significant symptoms of food addiction (YFAS 2.0 ≥ 6/11 and impairment/distress) and did not currently meet criteria for a substance use disorder or a restrictive eating disorder, and did not have a history of severe mental illness. Individuals who underwent weight loss surgery less than 3 years ago or were currently pregnant or breast feeding were also not eligible to participate in the treatment. A 50 USD gift card was provided to participants upon completion of all assessment measures. The study was approved by an FWA IRB at Atrium Health Wake Forest Baptist Medical Center and is registered as a clinical trial at https://clinicaltrials.gov (Identifier: NCT04373343). Two participants met eligibility criteria and were consented to participate in the treatment (accessed on 21 March 2023). 

#### 3.1.2. Quantitative Data

Participants completed a range of validated behavioral health measures (Table 1) that were administered electronically, pre- and post-clinical intervention. Data were maintained in a secure, HIPPA-compliant electronic database. Descriptive statistical analyses including means and standard deviations were calculated to examine the differences in both participants pre- and post-clinical intervention.

#### 3.1.3. Qualitative Data

Participants completed session-specific fidelity questionnaires at the end of every session to ensure that topics and interventions deemed to be critical were delivered in each session. They were also asked to rate the helpfulness, novelty, and quality of the information covered in each session on a scale of 1 to 7, with higher ratings indicating greater agreement of benefit. Embedded in the fidelity questionnaires were open-ended qualitative questions related to the participants’ experience of each session, including, “What was the most helpful topic discussed today?” “What was the least helpful topic discussed today?” and “Do you have any suggestions for changes?” Additional qualitative information was collected via observations and notations made by the leader during sessions, and quotes made by participants as noted by the leader.

#### 3.1.4. FACT Manual Treatment Approach

The content in the FACT manual included sixteen 90minute sessions that were delivered virtually using HIPPA compliant software (Version number: 43.10.0.27753) (Webex by Cisco; https://webex.com) (accessed on 21 March 2023) by a doctoral-level clinical psychologist who specializes in behavioral health and the treatment of obesity. Participants were also asked to journal all food intake and their personal risk level for addictive eating related to eating episodes in the Recovery Record digital application (https://recoveryrecord.com). This application allowed for, with participant approval, remote monitoring of food journaling by the psychologist leading the treatment so as to provide individualized feedback.

## 4. Result

### 4.1. Participants

Two participants were enrolled and treated with the FACT manual for this feasibility study.

Participant 1 was a 60-year old cisgender, heterosexual, white/non-Hispanic, college-educated, married female with severe food addiction, as defined by the YFAS 2.0. Participant 1 reported a history of frequent dieting behaviors throughout her life. She reported concurrent, monthly participation in a diet and lifestyle program, which included nutrition and exercise education at a local medical center.

Participant 2 was a 61 year-old, cisgender, heterosexual, white/non-Hispanic, college-educated, married female with severe food addiction as defined by the YFAS 2.0. Participant 2 disclosed a remote history of being underweight.

However, she reported a more recent history of being overweight, characterized by multiple extensive dieting behaviors and attempts to control her weight. She described herself as a “food addict” and reported active, concurrent participation in Overeaters Anonymous (OA), including attending meetings two times per week and engagement with an OA sponsor. Given that this is a psychological treatment study, it should be noted that Participant 2 was actively employed as a Licensed Clinical Social Worker (LCSW).

### 4.2. Qualitative Data

#### 4.2.1. Personalized, Harm Reduction Treatment Approach

Both participants expressed appreciation for the personalized, flexible approach of the FACT manual. They noted relief that the FACT treatment manual takes a harm reduction stance and does not require a lifelong commitment to abstaining from certain types or categories of food. Further, both participants repeatedly noted that they felt the FACT’s approach to identifying high-risk foods was personalized, considering the person and the context of the situation. Similarly, they reported benefit from learning how to employ specific, concrete strategies to identify their personalized risk for addictive eating and engage in subsequent informed decision making.

#### 4.2.2. FACT Manual Content: Helpful Content

*Psychoeducation:* Participants reported that they felt the psychoeducation provided in the manual was particularly helpful. They both indicated that the psychoeducation related to the neurochemistry and cycle of addiction, eating on a time-based schedule (every 3–4 h), benefits of protein intake, and urge-surfing were useful.

*Lifestyle Launch:* Both participants also reported that the preparation for the LL provided in session 8, and subsequent participation (sessions 9–12) in the LL were extremely helpful and distinct from past experiences attempting to manage their disordered eating behaviors. Participant 1 stated that she found value and comfort in the concept of using the 4-week LL to “rewire [her] brain”. Further, the participants reported surprise related to the ease with which they were able to comply with the request to limit their food intake to low-risk foods, particularly during the first 2 weeks of the Launch. Ease of implementation was attributed to having learned and practiced skills in the sessions prior to the LL.

*Food Expectations:* Despite an extensive history of dieting practices and attempts to manage addictive eating behaviors, both participants identified treatment content related to food expectations as highly valuable and novel. Participants reported new insight related to their expectations for foods, and how these expectations did not always match their actual experience of the food. More specifically, one participant reported expecting a food to be extremely tasty and comforting, but later found that the food was not actually as “good” or “comforting” as they expected it to be. In contrast, the other participant reported being surprised by how much she enjoyed and felt satisfied after eating low-risk foods. Challenging expectancies appeared to be important for treatment success.

Participants used their new understanding of their expectations for food to assess their risk-level and make lower risk choices. Evidence of this can be seen in a direct participant quote:

Through my years of addictive eating I had realized that sometimes my favorite (unhealthy foods) didn’t always live up to my expectations. I often thought, why am I eating this, it’s not worth the calories today. It was like I thought the next bite was going to be awesome and the way I remembered it. Food preparation (taste) is inconsistent. This brought it to the forefront and, hopefully, I’ll think it through more before indulging.

In subsequent sessions, where this participant participated in in vivo exposure to higher risk foods, she noted how her expectation of how much she would enjoy the food (i.e., a cupcake) was not met. She reported that eating the cupcake did not trigger her to continue to eat higher-risk sweet foods in the days following the exposure intervention, which reportedly would have been the case in the past. She attributed this accomplishment to new insight into her food expectations. In the subsequent week, she continued to make low-risk food choices.

#### 4.2.3. Weight Loss Goals

The desire for weight loss as an adjunct to the food addiction treatment was a common theme amongst both participants. For example, in session 2, participant 2 identified her most valued treatment goal as “weight loss”. Participant 1 also identified weight loss as a treatment goal; at the end of session 8, she reported, “I am frustrated that I have not lost weight. I am doing much better and making a lot of low-risk choices, but no weight loss”.

Similarly, both participants struggled to shift their emphasis on weight loss and a conceptualization of the treatment as a “diet” or a means by which to lose weight. They experienced difficulty applying the treatment intervention of rating foods as “high”- or “low”-risk based on their individualized vulnerability to addictive intake of the food. Rather, there was a strong tendency to rate a food as “high”- or “low”-risk based on their understanding of the potential of the food item to cause weight gain.

#### 4.2.4. Concurrent Treatments

Participant 2’s participation in OA during the FACT treatment provides unique insight into the possible challenges and/or advantages of concurrent treatments with FACT. The participant voiced awareness of times where the two treatments sent competing messages, particularly during sessions with exposure exercises (13 and 14), where she was encouraged to try a higher-risk food. During session 13, she reported some anxiety, as she selected exposure to a food item that was not consistent with OA recommendations. However, as she ate the item she reported that it did not trigger addictive eating symptoms as she expected, and reported, “it’s going better than I thought”, particularly with the support of eating slowly and mindfully.

Throughout the FACT treatment, participant 2 showed increased awareness of the possible negative impact of her rigid and self-depreciating thinking style. At times, the participant identified experiences in OA that may have reinforced this thinking style. She reported benefit from the cognitive restructuring component of FACT, as she was assisted in identifying and challenging the unhelpful cognition that “I am a failure” to “I had a few slips and can get back on track”. Following this intervention, the participant resumed adherence to a low-risk food plan.

There were also times where the information and skills gathered from OA participation appeared to benefit this participant. For example, after three sequential lapses into addictive eating, she decided to employ an OA recommendation of limiting her intake to “only green foods” for a period of time, which is also consistent with the LL recommendation of only eating low-risk foods for a period of time. This intervention was effective for her in preventing relapse.

Participant 1 participated in a lifestyle program at another local hospital concurrent with FACT treatment. This program was reportedly largely educational in nature, providing the participant with nutritional, exercise, and general health information. The participant described this program as a helpful adjunct to FACT treatment, as it increased her knowledge of basic nutritional principles and fitness/exercise.

#### 4.2.5. Expertise of Clinician

Participants both expressed the perceived benefit of having a trained mental health professional leading the intervention. Participant 2 reported that the clinician’s management of multiple slips related to high-risk foods through the use of cognitive restructuring was novel and helpful. Specifically, the clinician used cognitive restructuring to reduce negative and catastrophizing self-statements around off-plan eating behaviors to prevent a lapse from turning into a relapse. This intervention was notable for participant 2, as it was in contrast to her experience with OA. Furthermore, in session 12, this participant identified the “Therapist/leader recognizing a pattern that I did not recognize myself—major insight” as most helpful in that session.

#### 4.2.6. Treatment Tolerance and Progress

Both participants were able to adhere to the FACT treatment, attended all sessions, and reported increased confidence in their ability to manage addictive eating behaviors over the course of treatment.

### 4.3. Quantitative Data

*Fidelity Ratings.* Ratings across participants were consistent with all critical topics being covered in each session. Both participants rated the quality and the helpfulness of the information presented during the treatment as ≥5/7 across all sessions, except for the quality and helpfulness of Session 1 (Introduction to the Treatment) being rated as 4/7 by one participant. Modal ratings of the quality of the treatment content were 7/7 for both participants. Modal ratings of the novelty of the information presented were 5/7 and 7/7.

*Treatment Outcome Variables.* Both participants’ pre- and post-data are included in Table 2. Overall, both participants went from severe food addiction on the YFAS 2.0 at the beginning of treatment to endorsing no symptoms of food addiction at the post-assessment. General measures of eating disorder pathology also were reduced during treatment. Internalized weight stigma was reduced for one participant, but increased somewhat for the second participant. Participants reported reductions in symptoms of depression and anxiety across the treatment and no increases in alcohol or cannabis problems were observed. Finally, quality-of-life ratings and self-efficacy improved across multiple domains for both participants.

## 5. Discussion

The purpose of this article was to introduce the FACT manual, which applies a harm-reduction approach to address addictive food intake, and to disseminate qualitative and quantitative data from a pilot test of this treatment approach.

### 5.1. FACT Manual

Qualitative feedback from the participants highlighted beneficial aspects of the FACT manual. First, both participants reported that they found the harm reduction approach to be helpful and aided in learning new skills to manage high-risk foods. This is consistent with treatments for other addictive substances where an empirically supported treatment that does not require abstinence provides a wider range of treatment approaches that can be personalized to an individual’s goals [17,18,19,34]. This preference is supported by recent weight management technological applications (e.g., Noom) that also take a harm reduction approach. The FACT manual explicitly emphasizes that the world we live in makes it practically impossible to avoid high-risk hyperpalatable, ultraprocessed foods and offers an alternative way to reduce the harm associated with addictive patterns of intake. Participants also reported they enjoyed specific pieces of the manual including the psychoeducation component about the science of food addiction, the LL, and challenging food expectancies.

Qualitative feedback from the two pilot participants also identified two issues that the authors realize need to be addressed in future iterations of the FACT manual. The first was the issue of weight loss. Although the FACT manual clearly, and explicitly, emphasized the importance of *not* focusing on weight loss during the treatment, the participants expressed that it may not be feasible to have a treatment plan that does not discuss weight loss in some capacity. This was important feedback to consider, particularly within the context of understanding the potential presence of comorbid eating disorders when addressing participants’ weight loss goals. This observation may be associated with internalized weight stigma which can be a risk factor for maladaptive eating and eating disorder symptoms, as well weight-related outcomes, poorer mental and physical quality of life, greater perceived stress, less self-efficacy related to eating and physical activity, and exercise avoidance [35,36]. There is also evidence that obesity is associated with significant medical comorbidities, increased risk for mortality, and reduced quality of life, which may also motivate weight loss to improve health and comply with recommendations from medical providers [37]. Most likely, there is a complicated interaction between weight stigma and a desire for improved health that drives individuals’ goals for weight loss. Thus, future versions of the FACT manual need to consider participants’ weight loss goals while also addressing past experiences of weight stigma and the counterintuitive effects of internalization of weight stigma.

A second challenge that presented itself during the pilot test was the use of concurrent weight loss treatment interventions. Although, at first, the authors were concerned about the other treatments used by the participants, the feedback towards the end of the trial suggested that they felt the concurrent treatments were a helpful augmentation to the FACT manual treatment program. The authors will incorporate this into future versions of the manual so clinicians can receive guidance on how to manage participants who are utilizing other treatment interventions when participating in the FACT treatment protocol. Integrating this treatment approach with pharmacotherapy may be particularly important given the rising use of new-wave weight loss medications like GLP-1 agonists. There is some initial evidence that these medications may reduce addictive eating [38] and future research could consider evaluating the utility of combining pharmacological and psychosocial treatments.

The final qualitative feedback received from the participants was related to more general aspects of their experience and was positive. They reported they viewed the clinician as knowledgeable and effective as an interventionist. The participants were explicit in their appreciation for having a mental health expert as their group leader. They also reported that the treatment was easy to engage in and useful for managing food addiction.

### 5.2. Participant Outcomes

Pre- and post-intervention quantitative data also support the utility of the FACT manual. First, participants went from severe food addiction to no food addiction, as evidenced by their YFAS 2.0 scores. This result should be interpreted with caution as performance bias or social desirability bias may have influenced these scores. These results also could be initial evidence that a harm-reduction-based treatment approach may be effective in treating food addiction without requiring total abstinence or the opposite approach of promoting the intake of all foods in moderation. Second, there was a decrease in traditional disordered eating behaviors and a reduction in fasting and laxative use. Although concerns have been expressed that employing an addiction perspective, as is carried out in the FACT manual, may lead to a worsening of disordered eating [14], the pilot study did not support this concern. However, it is unknown how this would have impacted a person with a restrictive eating history. That being acknowledged, there is growing literature that treatment approaches that restrict intake of certain foods (without shame and blame about weight) do not seem to increase eating pathology and may even reduce it [39,40,41]. In sum, this pilot case study suggests that restriction of personalized high-risk foods through the lens of addiction in the FACT manual similarly did not cause an increase in harmful restrictive disordered eating. However, this is a small pilot study and further replication is needed, specifically in eating disorder samples and individuals with restrictive eating histories.

As mentioned in the discussion of the qualitative data, the FACT manual approach attempted to avoid explicitly promoting weight loss; however, participants reported that weight loss was a major motivational drive for both. One participant’s scores on internalized weight stigma went down over treatment, but the other person went up slightly. Given that weight loss promotion was not a goal sought by the FACT manual, the increase in weight stigma for one participant was surprising. It is possible that the omission of explicitly discussing the importance of personal weight loss motivations for engagement in the treatment may have been a missed opportunity to address internalized weight stigma and discuss the potential pros and cons of setting weight loss goals in conjunction with food addiction treatment. Thus, the combination of quantitative and qualitative data suggests that explicitly integrating participants’ personal weight loss goals safely and effectively into the FACT manual in a manner that does not further promote weight stigma should be an important future direction.

In addition to weight-related outcomes, there was also some evidence of beneficial outcomes in broader measures of psychopathology. Despite the treatment of mood/anxiety disorders not being explicitly addressed in the FACT manual, both participants reported improvements in this domain. The development of coping skills and mindfulness-based interventions over the course of treatment may have translated beyond the realm of addictive eating to the ability to address mood dysregulation more broadly. Also, there was no evidence that participants exhibited a transfer of addictive tendencies to alcohol/cannabis, which reduces concerns about this as an adverse consequence. Given that food addiction has been associated with worse psychiatric functioning and increased substance use [42,43], it is helpful that this treatment seems to lead to broader improvements in mental health functioning.

Consistent with this, participants also exhibited improvements in quality of life and improved self-efficacy across multiple domains. Food addiction is generally associated with lower quality of life and repeated failed attempts to alter food intake [11,44]. Thus, if future research demonstrates that this treatment improves overall quality of life for individuals with food addiction, there will be added clinical benefit. In addition, both participants had marked increases, from pre- to post-intervention, in self-efficacy to “stick with eating healthy foods”, “be physically active”, and “lose weight”. The ability of the FACT manual to increase self-efficacy may be important, as self-efficacy has been found to be an important predictor of behavior change [45].

Although all of these findings are promising, given that they are based on a sample of two individuals, all findings must be interpreted with caution. Further research with larger samples is warranted.

### 5.3. Limitations and Future Directions

There are a number of limitations of this study, but the primary one is obviously that the FACT manual was only pilot-tested on two participants, limiting the authors’ ability to draw conclusions from the findings. Also, participant 2 was a behavioral health provider which may have influenced her perception and feedback of the FACT manual. Although data was not gathered related to how this impacted her experience in the study, it is notable that as a behavioral health specialist she still sought treatment for her food addiction symptoms and reported benefit from the intervention. Another notable aspect of the participants was that both were cisgender, heterosexual, educated, and white/non-Hispanic. The experience of these two participants cannot be extrapolated to individuals from differing backgrounds and future studies need to emphasize recruitment of individuals from diverse backgrounds. Finally, this pilot test was an important step in identifying and addressing any potential problems in the manual that were not considered in the multiple iterations of the FACT manual. However, the FACT manual was designed to be delivered in a group therapy treatment approach. While the small number of participants allowed the clinician to work closely with participants and receive excellent feedback about the manual’s strengths and weaknesses, it did not allow the participants to experience a true group treatment approach.

Further research is clearly needed with larger sample sizes and individuals from more diverse backgrounds. Ultimately, research needs to be conducted to better understand, and incorporate, culturally relevant information related to food addiction. As future research is considered, it is important to note that the team that developed the FACT manual was also the research team who tested the manual on the two participants. Although the researchers aim to conduct a double-blinded randomized control trial with long-term follow-up, in the future, it is imperative that research also be performed outside of the laboratory that created the FACT manual to more independently determine the impact of the FACT manual on food addiction outcomes in clinical samples.

Despite these limitations, this initial pilot study of the FACT manual provides important information about the feasibility of applying a harm reduction treatment approach to address addictive patterns of food intake. Based on both qualitative and quantitative data, participants reported finding the treatment approach represented in the FACT manual beneficial, novel, and useful without strong evidence of adverse effects (e.g., increase in disordered eating, cross-transfer of addictive tendencies). The field of addiction treatment has broadly benefited from providing a number of empirically supported treatments (e.g., 12-step programs, harm reduction treatment, pharmacotherapy, motivational interviewing) that can be tailored to meet an individual’s specific goals and needs. The development of a similarly diverse range of treatment options to address addictive patterns of intake will also likely be beneficial.

## Figures and Tables

**Table 1:** Measurement tools administered pre and post FACT manual intervention.

Scale	Description and Scoring
Demographics	Created for the present study and included: age, sex, gender, race/ethnicity, education, and household income.
The Yale Food Addiction Scale 2.0 (YFAS 2.0 [6])	35-item self-report measure used to assess addictive eating behaviors related to ultra-processed foods. Items are scored to identify the number of endorsed symptoms of addiction and symptom count is associated with symptom severity (≥1 symptoms, no food addiction; 2–3 symptoms, mild food addiction; 4–5 symptoms, moderate food addiction; 6–11 symptoms, severe food addiction). To meet the threshold for food addiction, participants must have 2 or more symptoms plus clinically significant impairment or distress.
The Eating Disorder Diagnostic Scale (EDDS [25])	4 items from this self-report scale were selected to reflect diagnostic indicators of eating disorders associated with restrictive disordered eating. Specifically, participants indicated how many times (ranging from 0–12+ times) per month, for the past 3 months they had (1) vomited, (2) used laxatives or diuretics, (3) skipped at least 2 meals in a row, or (4) engaged in intense exercise to prevent weight gain or counteract the effects of eating.
Eating Disorder Examination Questionnaire Short form (EDE-QS [26])	12-item self-report measure assessing maladaptive or disordered eating behaviors over the past 7 days. Items scored on a 4-point Likert scale (0 = 0 days; 1 = 1 to 2 days; 2 = 3–5 days; 3 = 6–7 days). Total possible scores range from 0–36 with higher scores indicating greater maladaptive and disordered eating behaviors.
The Weight Self-Stigma Questionnaire (WSSQ [27])	12-item self-report measure of weight-related stigma including internalized weight stigma/self-devaluation and fear of enacted stigma. Items scored on a 5-point Likert scale (1–5) with possible scores ranging from 12–60, with higher scores consistent with greater experiences of weight-related shame and stigma.
The World Health Organization Quality of Life—BRIEF (QoL [28])	26-item self-report quality of life (QOL) questionnaire that assesses: physical health, psychological health, social relationships, and environmental. Items scored on a 5-point Likert scale (1–5) with scores ranging from 26 to 130 for each subscale with higher scores indicating better QOL.
Index of Sense of Self-Efficacy Scale (ISSES [29])	20-item self-report questionnaire that measures a participant’s confidence in their ability to take the necessary actions to accomplish a goal. Participants were asked to rate their level of confidence, on a range from 0–100% (with higher percentages associated with greater confidence), in their ability to “stick with eating healthy foods”, “be physically active”, and “lose weight”.
The Participant Health Questionnaire—9 (PHQ-9 [30])	9-item self-report scale measuring depressive symptoms that that align with the DSM-IV criteria for depression. Items are scored on a 4-point Likert scale (0–3), with a total possible score ranging from 0 to 27, with higher scores indicating greater symptoms of depression.
Generalized Anxiety Disorder 7 (GAD—7 [31])	7-item self-report screener that assess the presence and severity of symptoms of worry and anxiety. Items scored on a 4-point Likert scale (0 to 3), with a total possible score ranging from 0 to 21, and higher scores indicating greater symptoms of anxiety.
The Alcohol Use Disorder Identification Test (AUDIT [32])	10-item self-report measure assessing alcohol consumption, drinking behaviors, and alcohol-related problems. Items scored on a 5-point Likert scale (0–4), with a total possible score ranging from 0 to 40, and higher scores indicating greater symptoms of alcohol use disorder.
The Cannabis Use Disorder Identification Test-Revised (CUDIT [33])	8-item measure assessing problematic cannabis use. Item scores on a 5-point Likert scale (0-4), with a total possible score ranging from 0 to 32 and scores over 8 are indicative of hazardous cannabis use disorder.

**Table 2:** Participants’ quantitative scores pre- and post-FACT manual intervention.

Measurement Scale	Participant 1		Participant 2	
	PRE	POST	PRE	POST
YFAS 2.0	11	0	10	0
EDEQ-S	26	7	23	7
WSSQ	57	40	38	42
EDDS-Vomiting	1	0	0	0
EDDS-Laxative/diuretics	1	0	5	1
EDDS-Fasted	0	0	10	0
EDDS-Exercise	0	0	0	0
PHQ-8	21	13	7	3
GAD-7	14	5	8	3
AUDIT	0	0	2	1
CUDIT	0	0	0	0
QOL-PHYSICAL	31	44	44	63
QOL-PSYCH	31	38	38	50
QOL-SOCIAL	50	75	44	69
QOL-ENVIRO	94	88	44	69
SELF-EFFICACY EATING	44.75	58.25	26.25	55
SELF-EFFICACY PA	45	61.75	62.50	50
SELF-EFFICACY WL	42.50	59.50	20	52.50

Notes: Abbreviations—YFAS 2.0 (Yale Food Addiction Scale 2.0), EDEQ-S (Eating Disorder Eating Questionnaire-Short Form), WSSQ (Weight Self-Stigma Questionnaire), EDDS (Eating Disorder Diagnosis Scale), PHQ-8 (Participant Health Questionnaire-8), GAD-7 (Generalized Anxiety Disorder-7), AUDIT (Alcohol Use Disorder Identification Test), CUDIT (Cannabis Use Disorder Identification Test), QOL (quality of life), PA (physical activity), WL (weight loss).

## Data Availability

De-identified data will be available to researchers with a relevant research proposal on request.

## Supplementary Materials

The following supporting information can be downloaded at: https://www.mdpi.com/article/10.3390/bs14070557/s1, Figure S1: Food and Eating Addiction Test (FEAT); Table S1: FACT Manual Table of Contents.
