# Supplementary material for: The Food Addiction Clinical Treatment (FACT) Manual: A Harm Reduction Treatment Approach
Source: Behav Sci (Basel). 2024 Jul 1;14(7):557. doi: 10.3390/bs14070557 (PMC11273413; doi:10.3390/bs14070557)
Supplement: Supplementary file 1 [file behavsci-14-00557-s001.zip › behavsci-3060486-supplementary.pdf]

Supplementary Table S1 FACT Manual Table of Contents

Week 1: Phase 1 - Understanding Food Addiction, Introduction

- Introduction to group leaders and participants
- Group expectations
    i. Group as a safe place
    ii. Ways to benefit from group
- Program purpose
- Psychoeducation
    i. What is addiction?
    ii. The Food Environment
    iii. The Biopsychosocial Model
- Treatment Goal Setting
    i. The Lifestyle Launch
    ii. Abstinence versus Moderation? Neither—a Personalized Approach
- Food journaling
- Homework

Week 2: Phase 1 – Understanding Food Addiction -Personal Assessment

- Check in and Homework Discussion
- Food Journal Discussion
- Buddy Work
- Stages of Change
    i. Lapse vs. Relapse
    ii. Ambivalence
- Buddy Exercise: Pro/Con lists
- Food Expectations
- Homework

Week 3: Phase 1 – Understanding Food Addiction -Risky Foods

- Check in and Homework Discussion
- Common High-risk Foods
- An Individualized Approach – Beginning to Understand High and Low-Risk Foods
- Looking ahead: The Lifestyle Launch (Hitting the Reset Button)
- Buddy Exercise: High-Risk vs. Low-Risk Alternatives
- Homework

Week 4: Phase 2 – Skill Building - Risky Situations

- Check in and Homework Discussion
- Buddy Exercise – Looking at Food Journals
- Psychoeducation:
    i. Defining high-risk vs. low-risk situations
    ii. Rating your personal risk level – High-risk foods and high-risk situations
    iii. Managing risk and problem solving
- Homework

Week 5: Phase 2 – Skill Building - Coping Strategies

- Check in and Homework Discussion
- Introduction to Strategies to Manage Situational Risk Level
- Healthy and Unhealthy Coping Strategies
    i. Coping with food
    ii. Developing Healthy Alternative Strategies
    iii. Positive distraction
    iv. Relaxation breathing
- Buddy Exercise: New coping strategies
- Group Exercise: New coping strategies
- Homework

Week 6: Phase 2 – Skill Building - Identifying and Modifying Thought Patterns

- Check in and Homework Discussion
- Psychoeducation:

   i. Introduction to Cognitive Strategies
   ii. Understanding Cycles of Addictive Eating
- Identifying & Changing Expectations with Food
- Homework

Week 7: Phase 2 – Skill Building -Relationships and Communication

- Check in and Homework Discussion
- Introduction to Communication Strategies
- Making the Connection between Relationships and Eating Choices
- Navigating Social Situations Where There is Pressure to Eat
- Assertiveness Training
- Benefits of Being Assertive
- "Acting" Assertive
- Buddy Exercise: role playing Assertiveness
- Homework

Week 8: Phase 3 – Hitting the Reset Button: Lifestyle Launch (Preparing for Success)

- Check in and Homework Discussion
- Psychoeducation: Introduction to Phase 3 - Lifestyle Launch
- Group Exercise: Setting up your Food Environment for Success
- Buddy Exercise: Grocery Shopping & Meal Planning
- Homework

Week 9: Phase 3 – Hitting the Reset Button: Lifestyle Launch (Beginning of the 1st week)

- Check in and Homework Discussion
- Buddy Exercise: Identifying and reviewing foods that are high-risk for you
- Eating Out at Restaurants
- Understanding and Managing Cravings
   i. Withdrawal
   ii. Urge Surfing
   iii. Other tips for dealing with cravings
- Group Discussion: Healthy Coping with Challenging Emotions

- Saying Goodbye to a way of Life
- Patience and understanding
- Group Exercise: Why not stick to your old way of eating?
- Homework

Week 10: Phase 3 – Hitting the Reset Button: Lifestyle Launch (Beginning of the 2nd week)

- Check in and Homework Discussion
- Food Journal Review
- Revisiting Lapse vs Relapse
- Group Exercise: Navigating Restaurants
- Homework

Week 11: Phase 3 – Hitting the Reset Button: Lifestyle Launch (Beginning of the 3rd week)

- Check in and Homework Discussion
- Homework: Dining Out
- Mindfulness and Mindful Eating
- Group Exercise: Mindful Eating Activity
- Mindful Eating Tips
- Homework

Week 12: Phase 3 – Hitting the Reset Button: Lifestyle Launch (Beginning of the 4th and final week)

- Check in and Homework discussion
- Abstinence vs. moderation
- Post-Lifestyle Launch plan
- Buddy exercise: Identifying your Individualized Approach
- Introduction to Lifestyle Planning and Exposure Exercises
- Homework

Week 13: Phase 4 – Exposure and Maintenance

- Check in and Homework discussion
- Practicing Exposure
- Group Exercise: Eating Higher Risk Foods in Moderation
- Group Discussion of Exposure
- Revisiting your lifestyle plan
- Homework

Week 14: Phase 4 – Exposure and Maintenance

- Check in and Homework Discussion
- Group Exercise – Eating Higher Risk Foods in Moderation
- Group Discussion of exposure
- Introduction to outside food exposure
- Introduction to relapse prevention and coping
- What-The-Heck (WTH) Effect
- Homework

Week 15: Phase 4 – Exposure and Maintenance

- Check In & Homework Review: Outside Exposure
- Review of Relapse Prevention and the WTH Effect
- Buddy Exercise: Building your Toolbox
- Group Exercise: Preparing for life after group
- Homework

Week 16: Phase 4 – Exposure and Maintenance

- Check in: who has not completed the online post assessments?
- Group Exercise- Revisit Lifestyle Plan and Toolbox
- Group Exercise – Review of Treatment Goals
- Ceremony to Distribute Certificates & Mementos
- Post-treatment survey (if needed)
- Psychoeducation: Optional Post-Treatment Support
    - i. Optional: Booster post treatment sessions
    - ii. Optional: Plan post treatment support
    - iii. Optional: Further treatment options

Supplementary Figure S1

Food and Eating Addiction Test (FEAT)

Personal Risk

Is the food generally considered a high-risk or a low-risk food? Is it a naturally occurring food or a processed food? How risky is the food for you, personally? Is it one of your favorite foods? Is it a food that you have had difficulty controlling yourself when eating it in the past? Rate the riskiness of this food from 1 (low) to 9 (high).

1 2 3 4 5 6 7 8 9

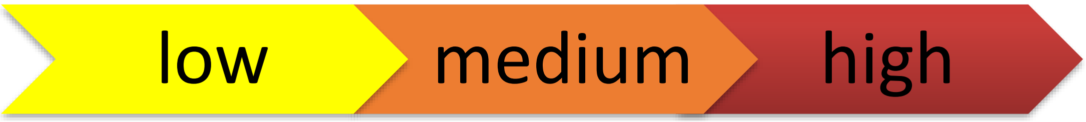

Situational Risk

How risky is the current situation? Remember the situation can mean many things. The situation could be related to the length of time since your last meal or how thirsty you feel. Also consider your emotional state—do you feel stressed, sad, angry, content, or happy? What is you physical location—are you in a place where it may be easy to overeat or pick the wrong choices—such as when you are alone, in your car, or at a restaurant? Rate the riskiness of this situation from 1 (low) to 9 (high).

1 2 3 4 5 6 7 8 9

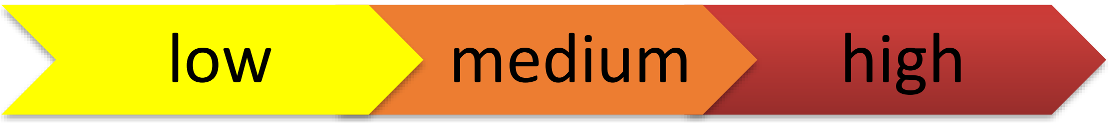

Overall Risk

To calculate Overall Risk, mix the colors of the rating that you assigned to personal risk and situational risk. Locate the resulting color on the band below to identify your overall risk.

yellow + yellow = low-risk
yellow + orange = low-moderate risk
yellow + red = moderate risk
orange + orange moderate risk
orange + red = high-moderate risk
red + red = high-risk

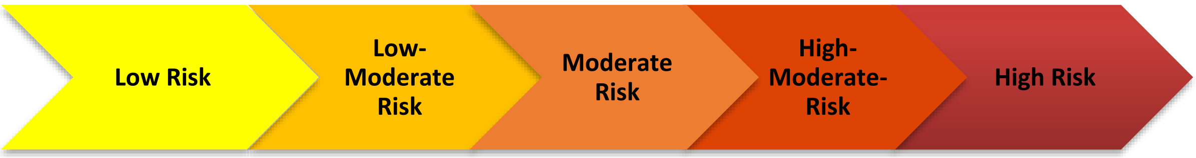

**Low-Risk**: Proceed mindfully	**Low-Moderate Risk**: Be aware of what is increasing your risk level. Monitor closely to make sure risk level does not increase	**Moderate Risk**: Consider all risk factors carefully, and if you proceed, proceed with caution and a clear plan	**High-Moderate Risk**: Employ strategies to reduce at least one risk factor (e.g., reduce stress, choose safer food)	**High-Risk**: There are multiple risks associated with this choice, reconsider this choice at this time.
